# Concentric Magnetic Structures for Magnetophoretic Bead Collection, Cell Trapping and Analysis of Cell Morphological Changes Caused by Local Magnetic Forces

**DOI:** 10.1371/journal.pone.0135299

**Published:** 2015-08-13

**Authors:** Chen-Yu Huang, Zung-Hang Wei

**Affiliations:** Department of Power Mechanical Engineering, National Tsing Hua University, Hsinchu City, Taiwan; RMIT University, AUSTRALIA

## Abstract

Concentric magnetic structures (ring and square) with domain wall (DW) pinning geometry are designed for biological manipulation. Magnetic beads collection was firstly demonstrated to analyse the local magnetic field generated by DWs and the effective regions to capture magnetic targets of size 1 μm. Primary mouse embryonic fibroblasts (MEFs) are magnetically labeled by internalizing poly (styrene sulfonic acid) stabilized magnetic nanoparticles (PSS-MNPs) and then are selectively trapped by head-to-tail DWs (HH DWs) or tail-to-tail DWs (TT DWs) to be arranged into linear shape or cross shape. The morphologies and the nuclear geometry of the cells growing on two kinds of concentric magnetic structures are shown to be distinctive. The intracellular magnetic forces generated by the local magnetic field of DWs are found to influence the behaviour of cells.

## Introduction

To manipulate microscale particles in heterogeneous suspensions can be significant for biomedical applications. The particles can be tagged to specific targets if functionalized with molecules on their surfaces, and assist in separating, sorting and assembling for high-throughput analysis or lab-on-a-chip (or μTAS) applications. [1–4] Techniques have been developed for accomplishing the manipulation of fluid-borne particles; for instances, microfluid flow,[5] magnetic,[6] electric,[7] optical,[8] or acoustic force.[9] Specifically, the exposure of magnetic force to biological systems cause little adverse effects, which make it an attractive approach for the biomedical experiments.

The essential principle of manipulating magnetic particle involves the magnetophoresis process driven by inhomogeneous magnetic fields, which should overcome the opposing forces like Brownian motion or viscous drag. In standard practices, high-gradient magnetic separators (HGMS) or NdFeB permanent magnet are usually used for magnetophoretic separation. However, these strategies usually concentrate targets into large aggregates, which have been said to impact the structure integrity of biological sample to cause physical damages and interfere with the recognition of fluorescent signals from the targets. Therefore, methods to modulate magnetic field at the microscale would be more favourable for biological manipulation. Previously, studies have revealed that the magnetization in soft ferromagnetic elements with high shape anisotropy would align along the boundary and be constrained geometrically; therefore, domain walls (DWs) would form to reduce the magnetostatic energy and act as tiny magnets to generate local stray fields. [10] Based on the concept, adjusting the design of magnetic elements during fabrication can be a potential strategy to modulate local magnetic field for the purpose of manipulation.

In this study, concentric magnetic structures (ring and square) with constricted geometry were designed to form stable DWs for biological manipulation. Magnetic beads collection was firstly demonstrated to analyse the local magnetic field generated by DWs and the effective regions to capture microbeads of size 1 μm. Mouse embryonic fibroblasts (MEFs) were magnetically labeled by internalizing PSS stabilized magnetic nanoparticles (PSS-MNPs) and were trapped by DWs individually. The trapped cells were cultured, and their morphologies were analysed to see the local magnetic field of DWs interact with the behaviour of cells.

## Materials and Methods

### Design and fabrication of the magnetic structure devices

Photolithography and e-beam evaporation were used to prepare concentric magnetic structures on glass substrate and followed by a lift-off technique. Since thick magnetic film tends to disassociate into multiple domains, five separate stacked thin film layers that each consist of 30 nm iron magnetic layer spaced with 10 nm titanium non-magnetic layer were used to assures the homogeneity of *N´eel* walls throughout the element and stabilize the quasi-uniform state of the magnetized square frame.[11] The aspect ratio of the length over the line width of the frame is chosen to keep the uniformity of the magnetization.

### Magnetophoretic bead collection by domain walls (DWs)

Microbeads (Dynabead MyOne Carboxylic Acid; Invitrogen) were applied to realize the effective capture region, magnetic gradient and magnetic force of the stray field generated by DWs in concentric structures.[12] The trajectories of the microbeads were tracked, and the displacements were analyzed. The velocities were decomposed into parallel component (v_∥_) and perpendicular component (v_⊥_) with respect to the reference axis determined from the trajectories (S1 Fig). For those microbeads that were far from the DWs, the motion should be mostly affected by Brownian diffusion forces while less affected by magnetic forces; therefore, both v_∥_ and v_⊥_ fluctuated. While for the microbeads that were attracted by the DWs, v_∥_ gradually increased and v_⊥_ did not change obviously. The average magnetic force in the capture zone and acceleration region were estimated by F_mag_ = ma_∥_ + F_drag_, where the hydrodynamic drag force (F_drag_) opposing the magnetic force (F_mag_), m is the mass of the particle and a_∥_ is the acceleration derived from v_∥_ versus time. The hydrodynamic drag force is expressed as F_drag_ = 6π*ηr*_*bead*_v_*∥*_, here η (= 10^−3^ Pa‧s) is the viscosity of the water, *r*_bead_ is the radius of the bead (= 0.525 μm). The relationship between magnetic force and the magnetic gradient of the magnetic microbead can be expressed as, ${\text{F}}_{\text{mag}}=\frac{{\text{V}}_{\chi }}{\mu_{0}}\left(\text{B}•\nabla \right)\text{B}$, where *μ*_*0*_ is the magnetic permeability in vacuum, and V is the volume of magnetic bead, and χ is the volumetric susceptibility (= 1.44).[13] Furthermore, since there are no time-varying electric fields or currents in the medium, ∇×B = 0 can be applied to obtain ${\text{F}}_{\text{mag}}=\frac{{\text{V}}_{\chi }}{2\mu_{0}}\nabla B^{2}$, [14] in which the magnetic force is related to the gradient of the square of magnetic induction *B*. We then study the gradient ∇B^2^ as an indirect but objective measurement of the magnetic force.

### Magnetic nanoparticles for magnetic cell labeling

The poly (styrene sulfonic acid) (PSS, M.W. = 75,000; Alfa Aesar) stabilized iron oxide magnetic nanoparticles (PSS-MNPs) were synthesized by directly precipitating iron oxide in the presence of PSS. PSS can self-assemble into micelles in the aqueous solution due to the hydrophilic group (–SO_3_H) and hydrophobic alkyl group. PSS micelles can serve as soft-template for MNPs to form inside them and protected MNPs against agglomeration by steric hindrance. [15, 16] Initially, 5 mL of PSS (30% w/v) was dissolved in 50 mL of distilled water under constantly stirring for 2 hours under nitrogen atmosphere to obtain PSS micelles. Thereafter, 10 mL of 1 mmol L^-1^ Iron (III) chloride (FeCl_3_·6H_2_O; SHOWA) and 10 mL of 0.5 mmol L^-1^ Iron (II) sulfate (FeSO_4_·7H_2_O, Aldrich) aqueous solutions were rapidly injected into the above mixture. Followed by heated up to 65°C, an additional 5 mL of 25% NH_4_OH was added, and the color of the reaction mixture turned black which indicated the formation of magnetite. The reaction was allowed to proceed for 5 h under vigorous stirring until the PSS stabilized magnetite nanoparticles were obtained. The resulting mixture was washed thrice with distilled water and was preserved for further application.

### Cell culture and magnetic labeling process

Mouse embryonic fibroblasts (MEFs) were obtained from the Bioresource Collection and Research Center (BCRC), Taiwan. (BCRC Number: M-EF001) MEFs were expanded and cultured in DMEM with high glucose supplemented with 10% heat-inactivated fetal bovine serum (30 min at 56°C) at 37°C and 5% CO_2_ environment. Prior to magnetic labeling, cells were seeded at a concentration of 10^5^ cells/well in 12-well culture plates to reach 90% confluence. After that, magnetically labeled cells were washed 3 times by centrifugation at 1500 rpm for 5 minutes and replaced to fresh PBS to remove nonspecific bounded MNPs. Consequently, the cells were concentrated by applying a magnetic field via a permanent magnet placed on the outer wall of the centrifuge tube, and were then put in a clean culture medium.

### Quantification of internalized nanoparticle by single cell magnetophoresis

The experimental procedure and setups for single cell magnetophoresis was previously reported.[17] Briefly, the magnetically labeled MEFs suspended in DMEM medium were submitted to a magnetic field gradient (∇B = 14 mT/mm) generated by a permanent magnet that was adapted to the apparatus of a microscope. As the magnetic force exerted by the magnetic moment of the MNPs inside a cell (m_cell_), F_mag, cell_ = m_cell_∇*B*, was balanced by the drag force F_drag, cell_ = 6π*ηr*_*cell*_ *v*_*cell*_ that opposed to the motion, the number of magnetic nanoparticles N inside a cell could be calculated by *N* = (36*ηR*_*cell*_ *v*_*cell*_)/(cM_s_ *D*_TEM_^3^∇*B*), where *R*_*cell*_ is the radius of cell (= 7.5 μm), *η* is the viscosity of the carrier liquid, *ν* is the cell velocity, *D*_*TEM*_ is the diameter of a magnetic nanoparticle from TEM images, and c is the ratio of the net magnetization of magnetic nanoparticles to their saturation magnetization M_s_ (= 0.98) from hysteresis loops.

### Prussian blue stain

MEFs cells were stained by Prussian blue stain to visualize the localizations of Fe_3_O_4_ directly and qualitatively analyze the magnetic labeling efficiency. Either magnetically labeled cells or control (without labeling) was fixed in 4% Glutaraldehyde (GA) for 30 min at -20°C. Prussian blue staining reagent 2% of potassium ferrocyanide and 6% HCl was mix 1:1 (v/v) and incubated with cells for 20 min and counterstained with nuclear fast red.

### Fluorescence imaging visualizes living cells and cytoskeleton

In order to visualize fluorescent signal produced by living cell, all cells were transfected with green fluorescent protein (GFP) gene.[18] After trapped by DWs, cells were stained with 1 μL of propidium iodide (PI) (stock = 10 μg/mL, Invitrogen), incubated in the dark for 15 min and sequentially analyzed by fluorescence microscope.[19] To visualize cytoskeleton of cells, cells were fixed in 4% paraformaldehyde (PFA) and then permeabilized for 30 sec with cold Acetone/Ethanol at -20°C. Primary F-actin antibodies (Sangon) were added (1:200 dilutions in 1% BSA) and incubated for 1 h at 37°C, and followed by anti-Rabbit-cy3 secondary (Sangon) antibody (1:300 dilution in 1% BSA solution) for 1 h at 37°C. The sample was then mounted in 90% glycerol. Images were acquired with an inverted fluorescence microscope (Olympus CKX41) which was equipped with blue, green & UV filters.

### Equipment and analysis

The mobile microbeads and cells that were attracted by the local magnetic force generated by DWs were recorded with a high resolution cooled colored CCD camera (Mshot) for 15–20 min at 5 frames per second. The image sequences were then imported into the public domain open source software, ImageJ (http://rsbweb.nih.gov/ij/), and the displacements of individual microbeads or cells were tracked via the plug-in “manual tracking”. The diameter of cells was determined by analyzing the photos of cells through the built-in measurement tool of the capture software (Mshot Digital Imaging System). Hydrodynamic size was measured using a dynamic light scattering size analyzer (DLS, Zetasizer Nano; Malvern), and Dispersion Technology Software was used for hydrodynamic size analysis. The particles were examined with a transmission electron microscope (TEM, HT7700TEM; Hitachi) and a total of about 1000 particles from several TEM images were analyzed to obtain particle size in the dry state. The size distribution could be fitted using the log-normal function, [20] where the characteristic diameter D_0_ and the polydispersity parameter (or standard deviation) σ can be obtained. (Detail in S1 File) The value of average particle diameter D_TEM_ can be estimated by taking polydispersity σ into account, [21] which is 11.18 nm.

## Results and Discussion

**Fig 1:**
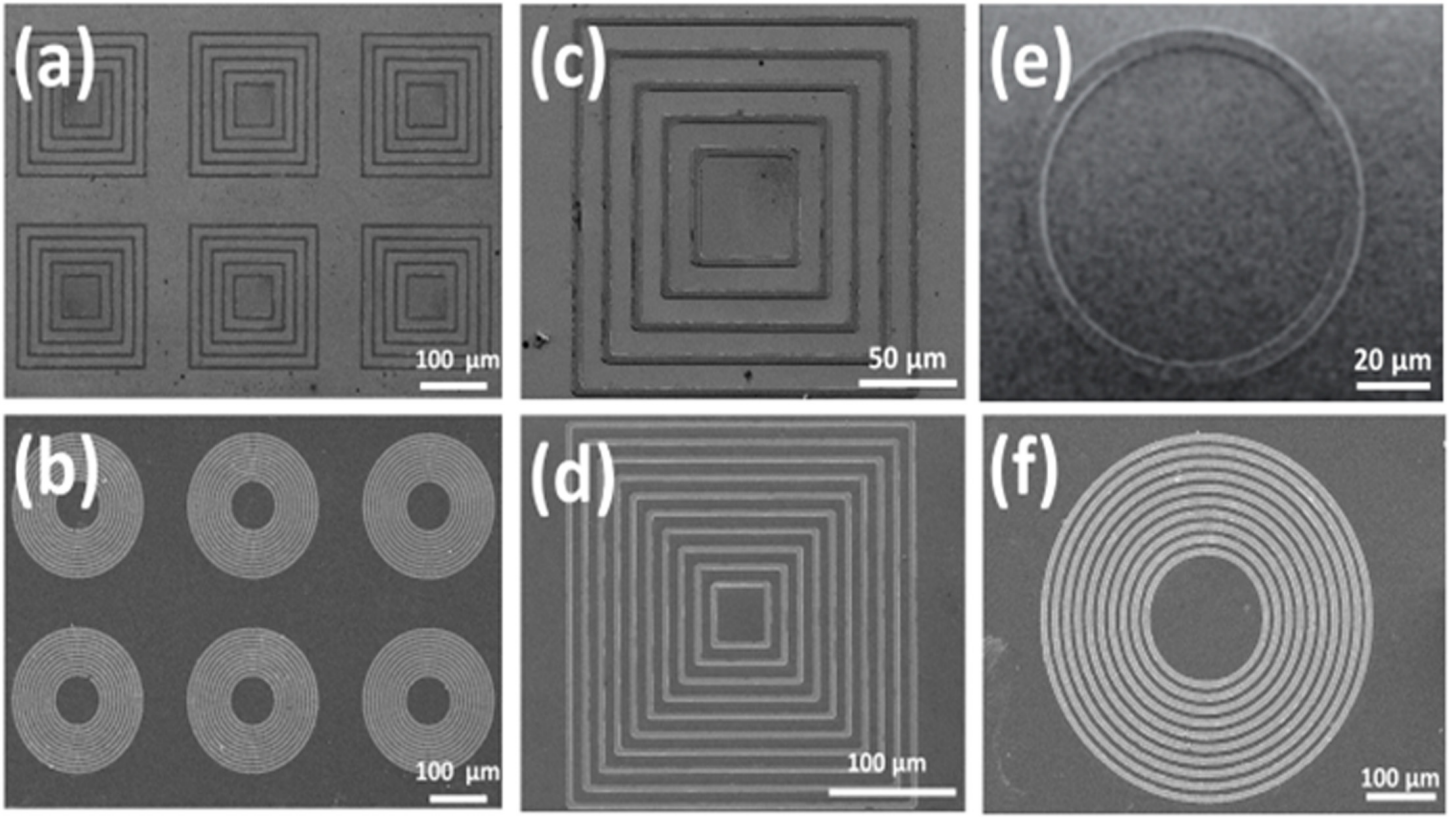
Representative topographic SEM images of magnetic concentric structures. (a) and (b) are array of magnetic structure (c) Single five period's concentric squares (d) Single 10 period's concentric squares (e) Single ring (f) Single 10 period's concentric rings. For square elements, the inner square has a side length of 50 μm, the line width is 4 μm and interval between lines is 12 μm. For ring elements, the inner ring has a diameter of 100 μm, the line width is 4 μm and interval between lines is 4 μm.

**Fig 2:**
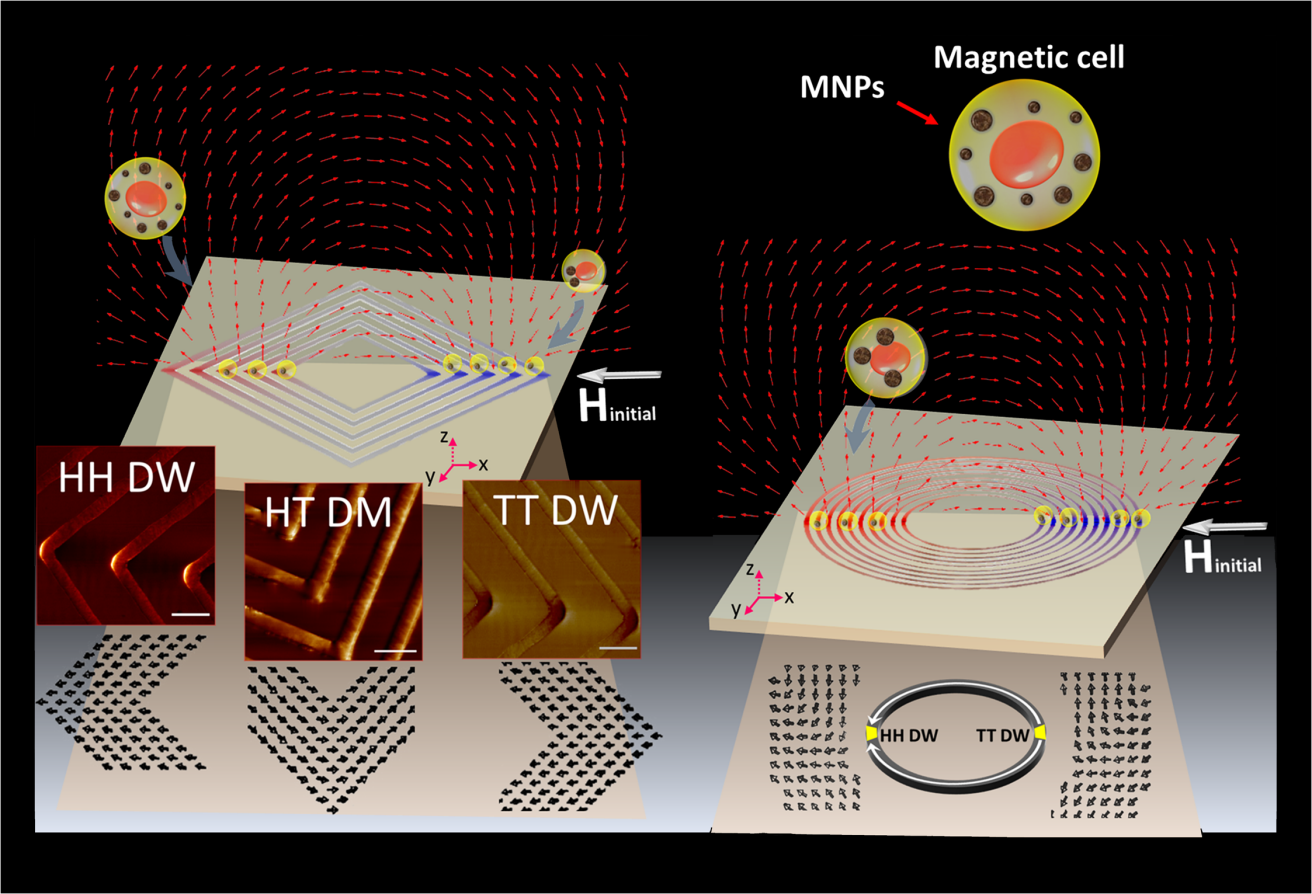
Schematics of cell trapped by the local stray fields generated by the domain walls of concentric squares and concentric rings. The insets are magnetic force microscopy (MFM) images of concentric squares at remanent magnetic states after an external magnetic field applied along one diagonal axis. The spin configurations symbolize as arrows. HT DWs, TT DWs, and HH DWs represent head-to-tail domain walls, tail-to-tail domain walls, and head-to-head domain walls, respectively. MNPs represent magnetic nanoparticles. Scale bars represent 10 μm.

Two kinds of concentric magnetic structures (ring and square) with different periods were designed to form stable DWs, which representative SEM images are shown in Fig 1. The concept of utilizing the local stray fields generated by DWs to attract magnetic targets (cells) is shown in Fig 2. To create stable DWs, a magnetic field (3000 Oe) was applied in the in-plane direction (x-y plane) of the concentric structures to saturate the magnetization and subsequently receded towards zero field, indicated as H_initial_. For concentric squares, as the H_initial_ was applied along one of the diagonal axes, the resultant magnetization was aligned locally and constricted at the corners of the squares due to geometric anisotropy. As shown in the spin configuration of Fig 2, head-to-tail DWs (HH DWs), tail-to-tail DWs (TT DWs) and head-to-tail DWs (HT DWs) are simultaneously appeared in concentric squares, while HH DWs/TT DWs appeared at the corners parallel to H_initial_ and HT DWs located at the others that perpendicular to H_initial_.[22] For concentric rings, after H_initial_ applied, the resultant magnetization would align at the border of ring, and two HH DWs and TT DWs were oppositely formed at two ends of ring. From the MFM images accompanied with the spin configurations, HH DWs/TT DWs showed either positive or negative magnetic poles characteristics with wider distribution; while HT DWs consisted of 90° Neel type domain and accumulated both positive/negative poles in a narrower distribution.

**Fig 3:**
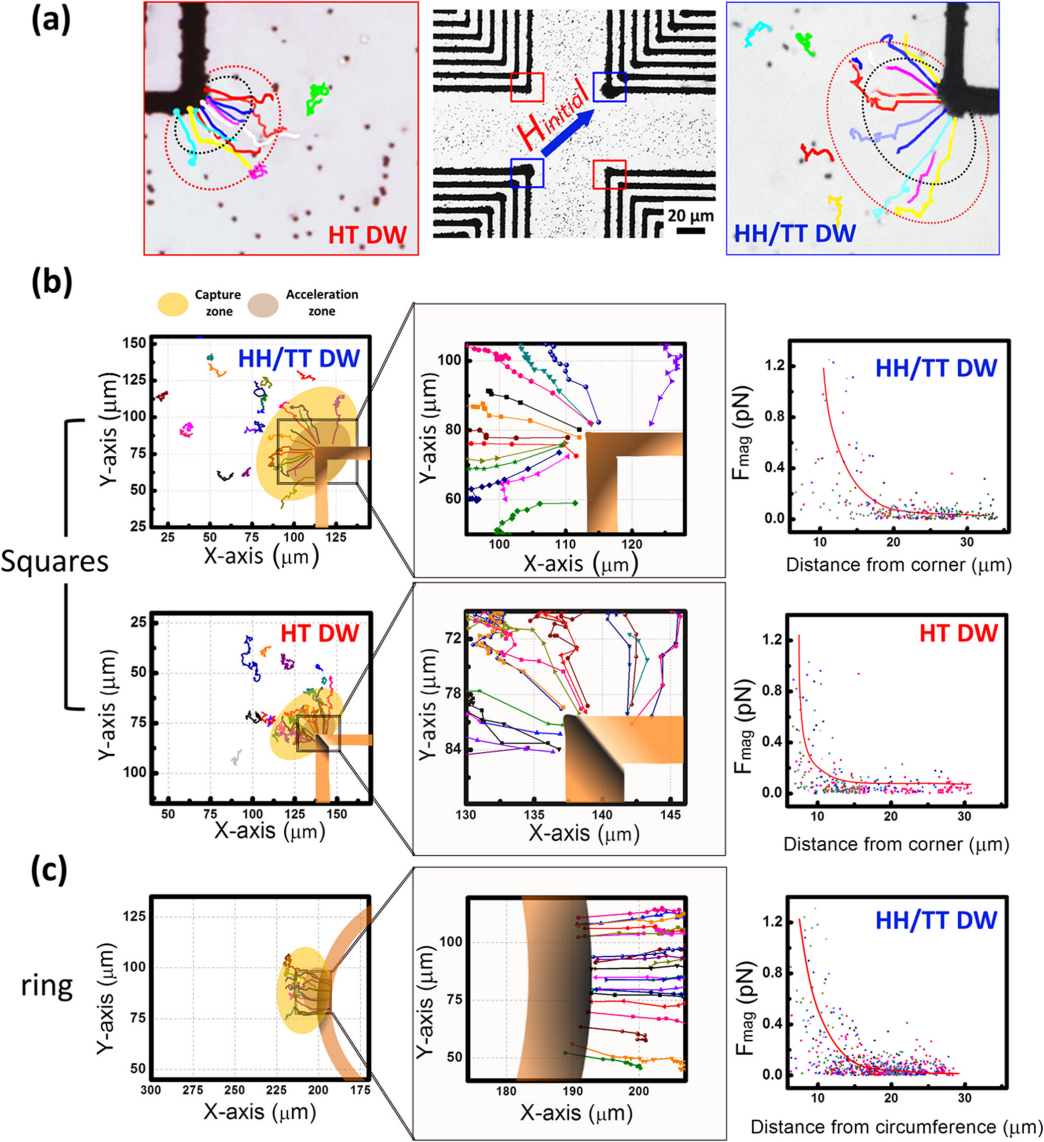
Magnetophoretic bead collection by DWs. (a) The picture of the microbeads captured by DWs. Regions that microbeads underwent magnetophoresis (red) or accelerated toward DWs (black) are highlighted. (b) Trajectories of microbeads trapped by DWs of square elements (c) trajectories of microbeads trapped by DWs of ring elements. The areas that microbeads accelerated toward DWs were separated in a magnified plot. Magnetic force derived from F_mag_ = ma_∥_+F_drag_, where the hydrodynamic drag force (F_drag_)opposed the magnetic force (F_mag_).

Suspended magnetic beads attracted by DWs were tracked and analyze to realize the local magnetic fields generated by DWs and further, to realize the effective capture regions. The motion of the microbeads would be affected by Brownian diffusion forces; however, if attracted by the local magnetic force of DWs, parallel component of velocity (v_∥_) would gradually increased (Detail in experimental section and S1 Fig). Since the magnetization of HT DWs is approximately in a flux-closure loop, the local stray field produced by HH DWs or TT DWs are much stronger than HT DWs; therefore, more microbeads can be collected by the HH/TT DWs than HT DWs in concentric squares, as can be visualized in Fig 3A. For concentric squares, the corresponding maximum magnetic field gradient (∇B^2^) near HH/TT DWs and HT DWs of concentric squares are 2.08 T^2^/m and 1.3 T^2^/m, while the HH/TT DWs of ring have maximum ∇B^2^ of 1.7 T^2^/m. The effective capture region to collect microbeads is larger for HH/TT DWs (16.3 μm) than that for HT domain (9.95 μm) for concentric squares, while the region for concentric rings have capture region of 12.55μm (Fig 3B and 3C). The modulation of local fields by forming HH/TT DWs in concentric squares can collect microbeads effectively which can be further demonstrated in S1 video. The forces exerted on individual microbeads while collection were estimated to be in a range of 0.57 to 0.65 pg were gentle enough for biomolecules; sample with dilute concentration can be bound to microbeads and be isolated and concentrated to DWs area for in-situ analysis.

**Fig 4:**
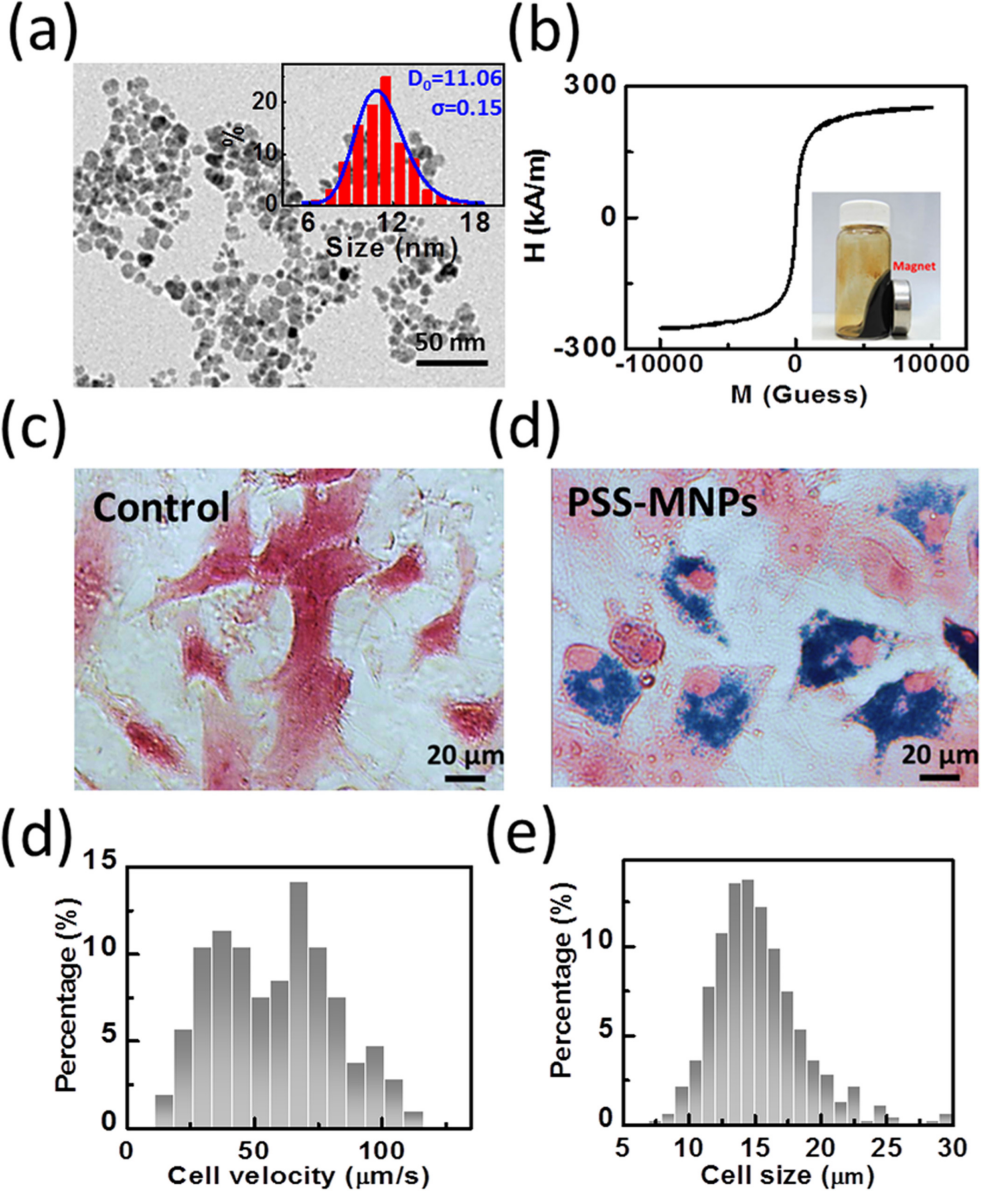
Characterization of PSS stabilized MNPs (PSS-MNPs) and single cell magnetophoresis. (a) TEM images and the size distribution of PSS-MNPs. The curve is a log-normal fit to the histogram. (b) The magnetization versus magnetizing field curve (M-H curve) at room temperature of PSS-MNPs. (c) Optical microscope images of control and cell magnetically labeled by PSS-MNPs. (d) Histogram of the cell velocity distribution derived from magnetophoresis experiment. (e) The size of cells determined by the picture of cells captured by the optical microscope.

Primary mouse embryonic fibroblasts (MEFs) were magnetically labeled via internalization of PSS-MNPs through endocytosis. The average size (D_TEM_) of PSS-MNPs was 11.18 nm obtained from TEM image in Fig 4A. and no hysteresis suggest superparamagnetic behavior (Fig 4B). After Prussian blue staining, cells internalized PSS-MNPs showed blue deposits in the cytoplasm while control cells did not (Fig 4C). The number of internalized MNPs was then quantified by obtaining the constant cell velocity in magnetophoresis experiment. In Fig 4D, the velocity is 54.7 ± 18.8 μm/s and the average cell radius of 7.5 μm (Fig 4E); therefore, the number of MNPs per cell could be estimated to be (4.11 ± 1.41)× 10^6^/cell, which is equal to 10.90 ± 3.75 pg iron/cell.

**Fig 5:**
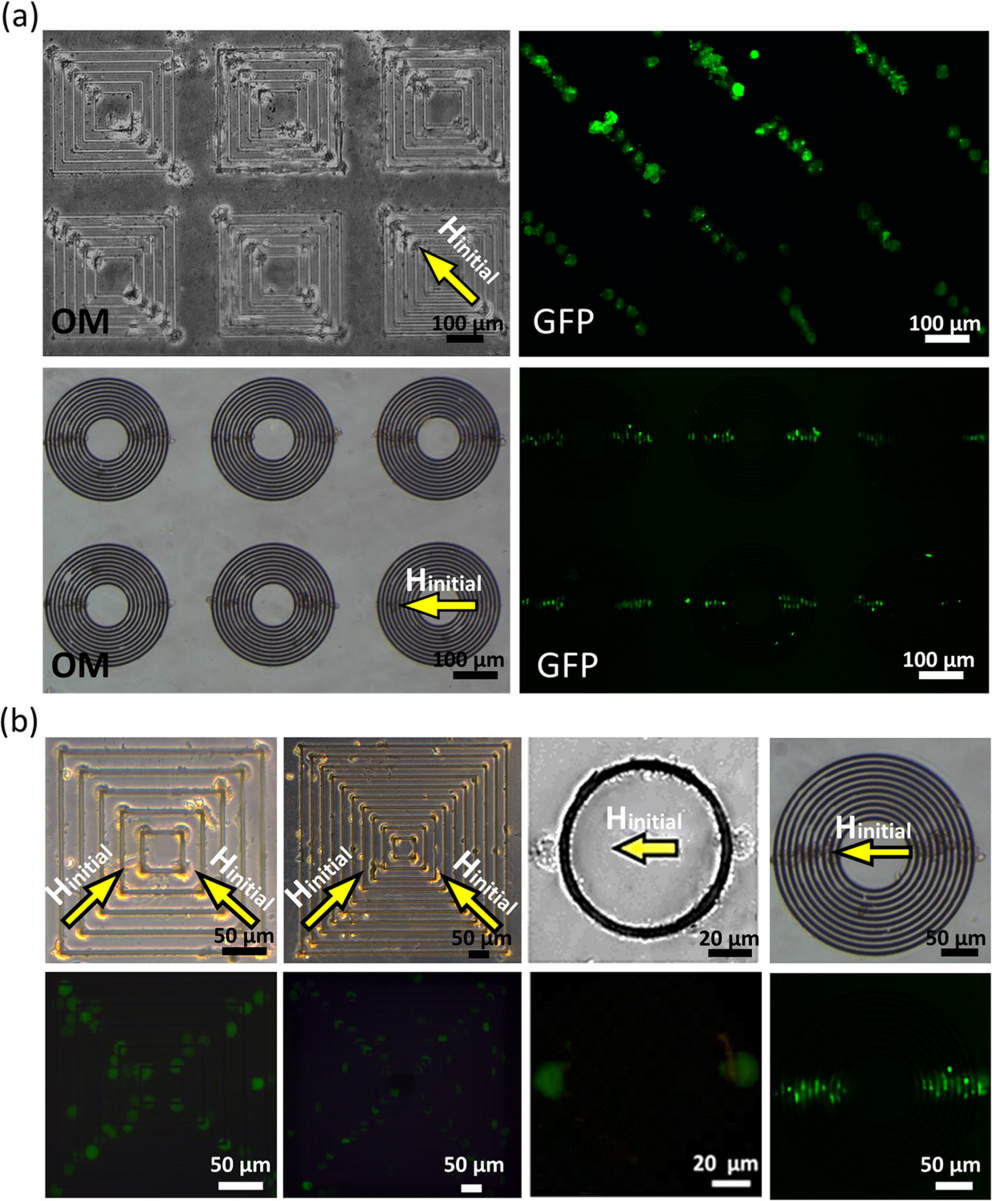
Optical microscope images and fluorescence images of the cell trapped by DWs of magnetic ring/squares structures after initial magnetic fields (H_initial_) applied. (a) Arrays of cells were trapped to arrange into the linear shape on magnetic ring/squares structures. (b) Cells trapped by magnetic rings/squares with different periods. Note that after two independent H_initial_ applied, cells could be arranged into cross-shape on concentric squares.

**Fig 6:**
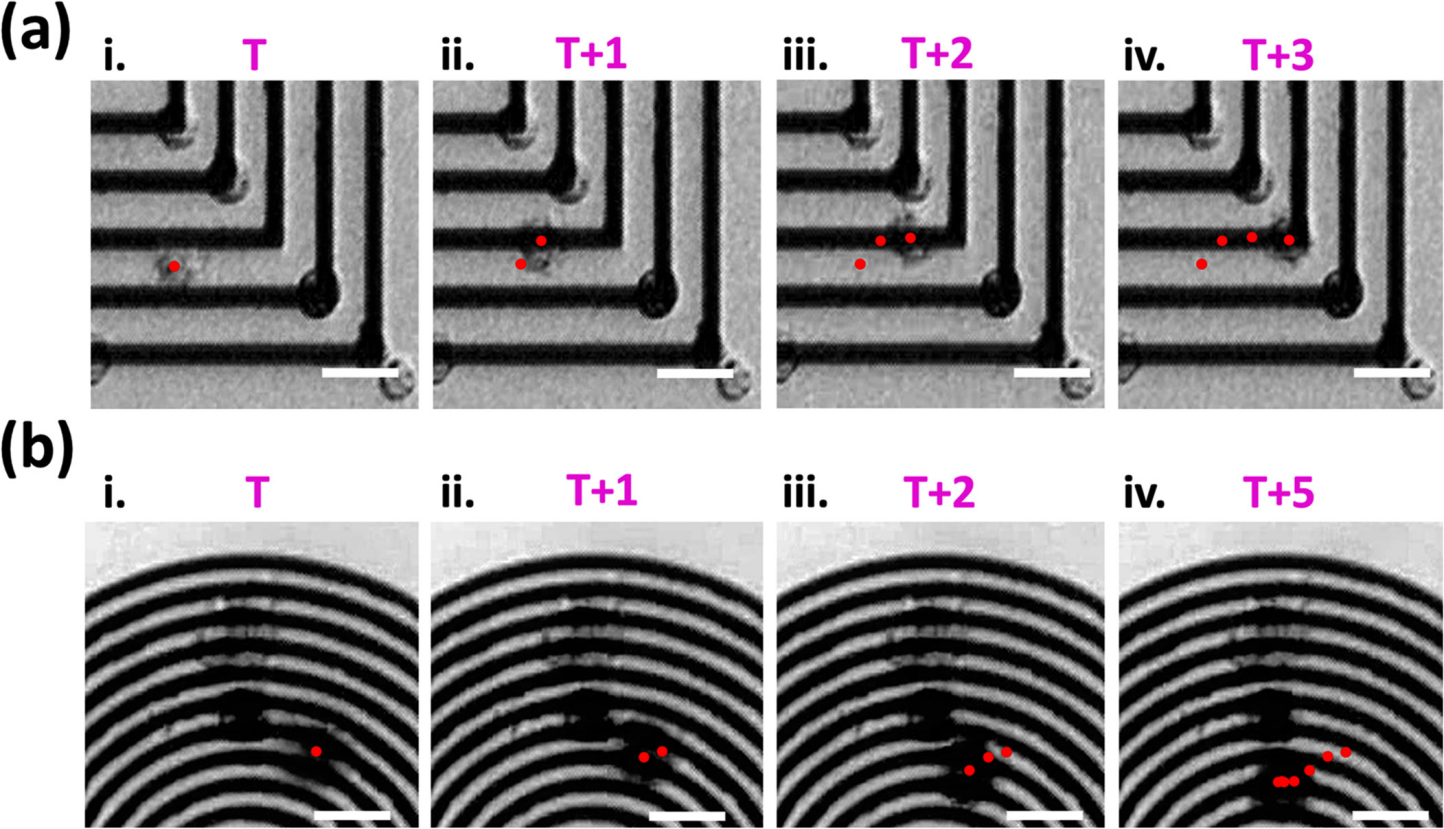
Sequential images show the moving paths of magnetically cell trapped by DWs. (a) Concentric squares (b) concentric rings. Scale bars represent 20 μm. The centroid positions of the cells are denoted with the dots.

The local magnetic fields are broader and larger in the vicinity of HH/TT DWs then HT DWs, which can selectively trap cells near HH/TT DWs. As shown in Fig 5A, the GFP-expressed MEF cells were trapped by HH/TT DWs instead of HT DWs and arranged orderly at remanent state after H_initial_ was removed. Magnetic structures with different periods could achieve similar results, as see in Fig 5B. Specifically, for concentric squares, if a subsequent magnetic field was applied along one diagonal axis to create HH/TT DWs after the previous cells have been trapped and attached along the orthogonal diagonal axis, another group of cells could be trapped to form cross-shape. Cells attracted to the DW of concentric structures were recorded by camera (S2 and S3 videos) and the sequential images of a moving cell are shown in Fig 6. The field gradients were larger in the vicinity of DWs and the cells will eventually accelerating toward the magnetic frames as getting closer to it. Cell velocities were then be determined by the trajectories of cells that were attracted by the DWs and the trapping force would be estimated to be about 2.8 to 7.9 pN for square elements and 1.1 to 3.5 pN for ring elements. The order of piconewton that microbeads and cells experienced was similar to that of previous literature, [23,24] and this show the possibility of utilizing geometrically constrained DWs as an alternative approach to separate, capture.

**Fig 7:**
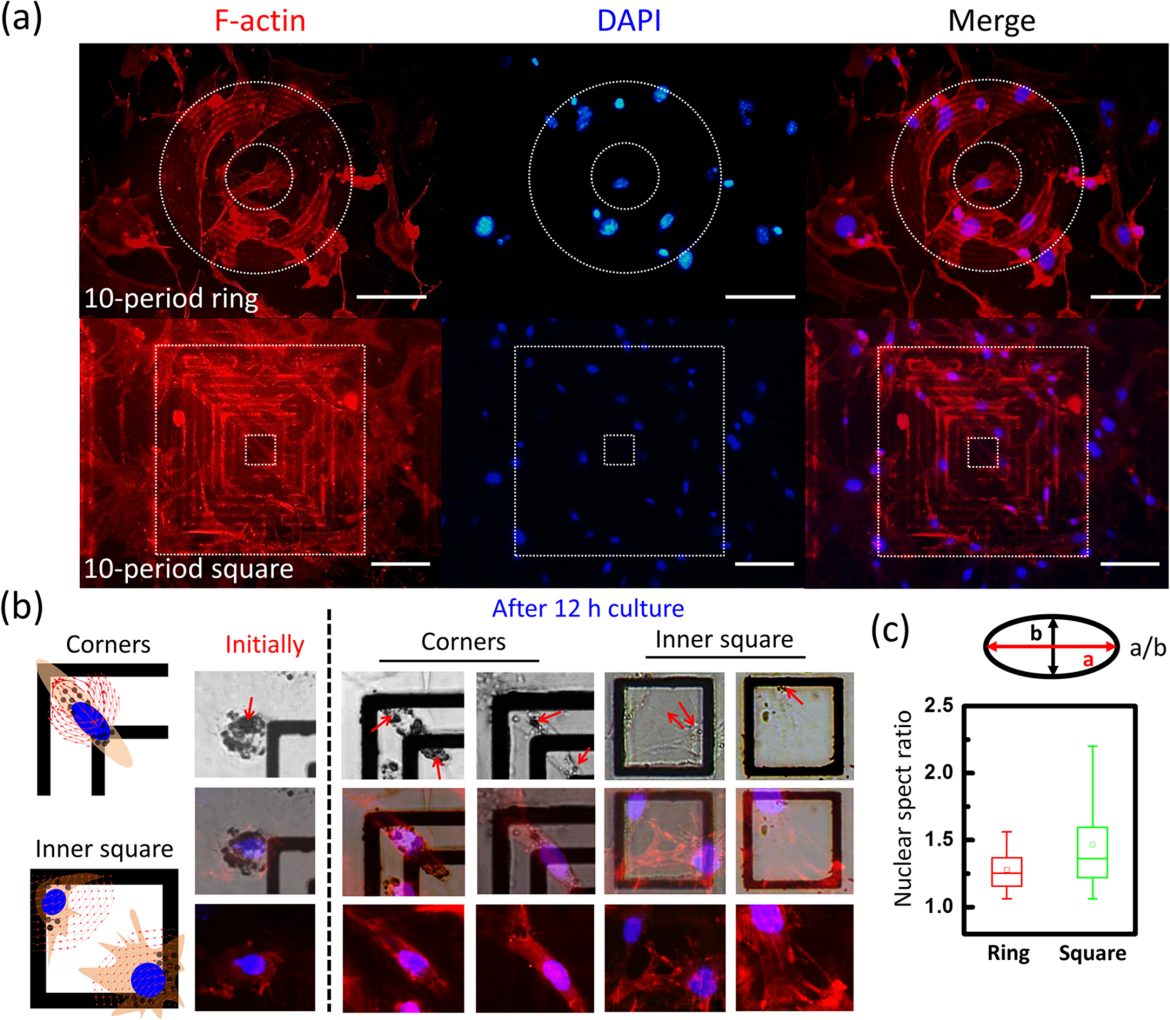
Fluorescence images, cell morphologies and nuclear aspect ratio of the cells growing on magnetic structures. (a) Representative fluorescence images of the trapped cells after culture for 2 days. Actin stained by anti-F-actin-cy3 (red) and nuclei stained with DAPI (blue). Dash lines indicated the inner and outer frames. (b) Representative morphologies of the cells initially attach to the substrate and after culture for 12 h. The intracellular localizations of MNPs are denoted by arrows optical images. (c) Box plot show the nuclear aspect ratio of cells growing on concentric rings and concentric squares. Scale bars represent 100 μm.

To adopt the concentric structure for cell analysis, the trapped MEF cells were kept cultured with the structure for 2 days to see the growth of magnetic cells influenced by the local magnetic field generated from DWs. In Fig 7A, filopodia-like protrusions and cell–cell contacts appear; the cells prefer to spread out as grow on ring elements but tend to stretch and elongate on square elements. As grow on concentric squares, cells were round in shape in the initial 2 h and then elongated and stretched out to the adjacent corners after 12 h of incubation (Fig 7B). The intracellular vesicles that contain MNPs were distributed randomly in the initial 2 h but located near DWs. For cells sedimented on the inner corners, they showed less preference to grow in a particular orientation. Recent reports have shown that the growth of cells internalized with magnetic nanoparticles can be modulated by external magnetic fields. [25, 26] The changes of the cell morphology may be contributed to the magnetic flux and the local magnetic force generated from the neighboring DWs. Further, the stretch of cell was coupled with nuclear deformation due to the compressive and lateral pulling stress from actin fibers transmitted to the cell nucleus; [27] therefore, the nuclear aspect ratios of cells growing on square elements were found to be larger than those growing on the ring (Fig 7C).

In this study, the results shows the strong localized magnetic fields and magnetic gradients from HH/TT DWs can drag specific targets from the biological sample, which is beneficial for separating, sorting and assembling biomolecules for further analysis. In contrast to the traditional method based on utilizing permanent magnets with the size of centimeter or millimeter that concentrate the sample into large aggregates, the proposed method manipulates targets in microscale. Since large aggregates have been said to cause physical damages to cells and interfere diagnosis, the present study shows that the patterned magnetic concentric structures can be used as an alternative strategies to capture and retrieve individual captured cells for downstream analyses. Besides, the proposed magnetic structures can actively trap and concentrate the rare targets which are particularly important for the development of implantable sensors in microfluidic channel in lab on a chip system to increase the detection and promote early diagnosis and screening.

## Conclusion

In summary, concentric magnetic structures with domain wall pinning geometry were utilized to collect magnetic beads and trap magnetically labeled cells. The effective regions to capture magnetic beads, the strength of magnetic forces and field gradient generated by DWs were compared. The local magnetic fields are broader and larger in the vicinity of HH/TT DWs then HT DWs, which can selectively trap cells near the region forms HH/TT DWs. The morphologies and the nuclear geometry of the cells growing on two elements were shown to be distinctively different. The intracellular magnetic forces generated by the local magnetic field of DWs are found to influence the behavior of cells.

## Supporting Information

S1 Fig Analysis of trajectories and velocities of microbeads. Trajectories and velocities of beads that move randomly (a) and captured by HH/HT DWs and HT DWs (b), (c). The velocities were decomposed into parallel component (v_∥_) and perpendicular component (v_⊥_) based on the reference axis that determined from the trajectories (red line). V_∥_increased steadily indicated acceleration.
(TIF)

S2 Fig Hydrodynamic size distribution of bare MNPs and PSS-MNPs. Bare MNPs indicate MNPs synthesized without poly (styrene sulfonic acid) (PSSA). The MNPs were mixed with deionized water (v/v = 1/2000) before the measurement.
(TIF)

S3 Fig TEM images of bare MNPs and PSS-MNPs. (a), (c) pictures of bare MNPs (b), (d) pictures of PSS-MNPs.
(TIF)

S4 Fig Sedimentation tests of the bare MNPs and PSS-MNPs. Bare MNPs and PSS-MNPs diluted in deionized water (DI water) and DMEM media for different time periods (0, 15 min, 1 h, and 24 h). The MNPs were mixed with (v/v = 1/100).
(TIF)

S1 File Particle size determined from Transmission Electron Microscopy (TEM). (DOCX)

S1 Video Microbeads collected by concentric squares. (ZIP)

S2 Video MEF cells trapped by concentric squares. (ZIP)

S3 Video MEF cells trapped by concentric rings. (ZIP)
