# Supplementary figures and images for: Concentric Magnetic Structures for Magnetophoretic Bead Collection, Cell Trapping and Analysis of Cell Morphological Changes Caused by Local Magnetic Forces
Source: PLoS One. 2015 Aug 13;10(8):e0135299. doi: 10.1371/journal.pone.0135299 (PMC4536140; doi:10.1371/journal.pone.0135299)

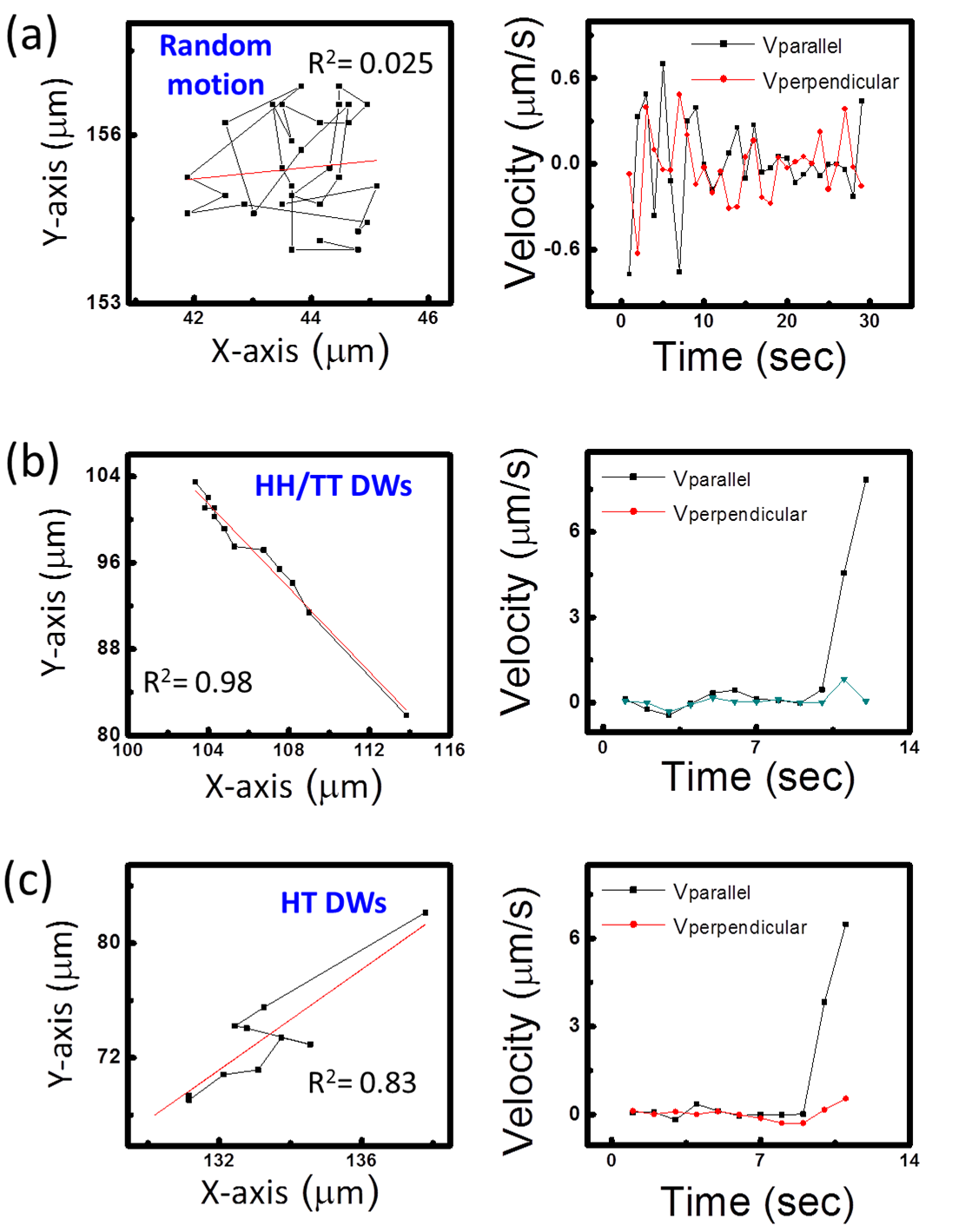

Supplement: S1 Fig — Trajectories and velocities of beads that move randomly (a) and captured by HH/HT DWs and HT DWs (b), (c). The velocities were decomposed into parallel component (v∥) and perpendicular component (v⊥) based on the reference axis that determined from the trajectories (red line). V∥increased steadily indicated acceleration. (TIF) [file pone.0135299.s001.tif]

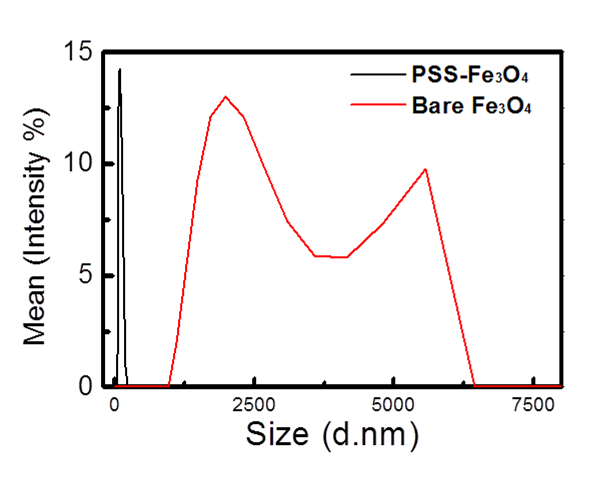

Supplement: S2 Fig — Bare MNPs indicate MNPs synthesized without poly (styrene sulfonic acid) (PSSA). The MNPs were mixed with deionized water (v/v = 1/2000) before the measurement. (TIF) [file pone.0135299.s002.tif]

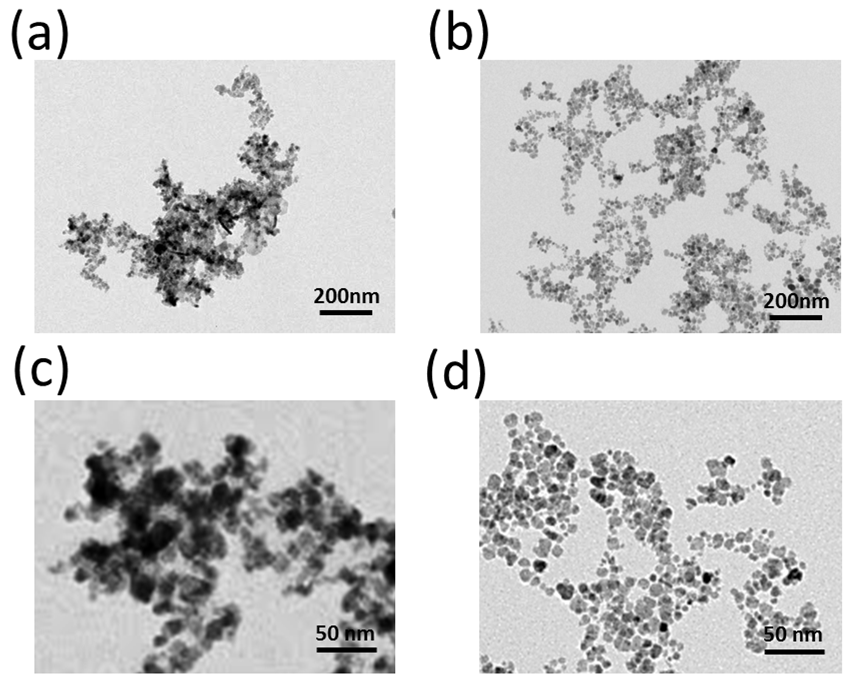

Supplement: S3 Fig — (a), (c) pictures of bare MNPs (b), (d) pictures of PSS-MNPs. (TIF) [file pone.0135299.s003.tif]

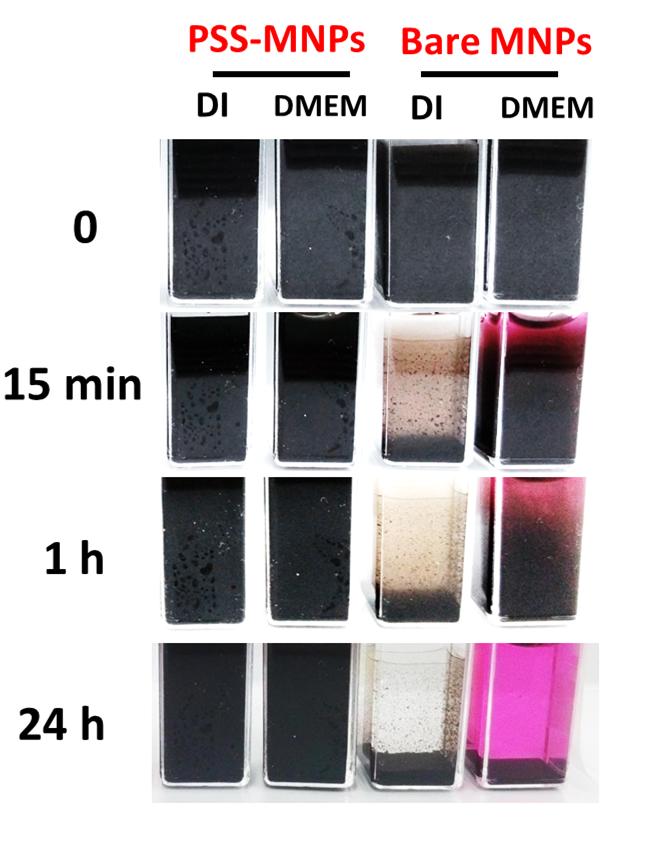

Supplement: S4 Fig — Bare MNPs and PSS-MNPs diluted in deionized water (DI water) and DMEM media for different time periods (0, 15 min, 1 h, and 24 h). The MNPs were mixed with (v/v = 1/100). (TIF) [file pone.0135299.s004.tif]
