# Supplementary material for: Concentric Magnetic Structures for Magnetophoretic Bead Collection, Cell Trapping and Analysis of Cell Morphological Changes Caused by Local Magnetic Forces
Source: PLoS One. 2015 Aug 13;10(8):e0135299. doi: 10.1371/journal.pone.0135299 (PMC4536140; doi:10.1371/journal.pone.0135299)
Supplement: S1 File — (DOCX) [file pone.0135299.s005.docx]

Supporting Information: Magnetophoretic bead collection, cell trapping, and cell morphology changes induced by local magnetic force using concentric magnetic structures

### Particle size determined from Transmission Electron Microscopy (TEM):

Literatures have shown lognormal law to be appropriate for analyzing particle size distribution analysis of spherical particles. [1, 2, 3] Probability density P(D) can be used to obtain the characteristic diameter D_0_ and the polydispersity parameter (or standard deviation) σ can be written below: [4]

$\text{P(D) =}\frac{1}{\sqrt{2\pi\sigma D}}\text{exp}\left\{\text{-}\frac{\left[\ln\left(D/D_{0}\right)\right]^{2}}{2\sigma^{2}}\right\}$ (S.1)

The respective value of D_0_ and σ are found to be 11.06 nm and 0.15 form size distribution obtained from TEM images. The average size D_TEM_ can then be estimated by taking polydispersity σ into account,$\text{D}_{\text{TEM}}\text{ =D}_{0}\exp\left(\sigma^{2}/2\right)$, which is 11.18 nm.

**REFERENCES**

[1] Konstantinović Z, del Muro MG, Varela M, Batlle X, Labarta A, (2006) Particle growth mechanisms in Ag–ZrO_2_ and Au–ZrO_2_ granular films obtained by pulsed laser deposition. Nanotechnology 17: 4106-4111.

[2] Lavorato GC, Jr EL, Tobia D, Fiorani D, Troiani HE, Zysler RD, et al. (2014) Size effects in bimagnetic CoO/CoFe_2_O_4_ core/shell nanoparticles. Nanotechnology 25:355704 (9pp).

[3] Paula FLO, Silva GJda, Aquino R, Depeyrot J, Fossum JO, Knudsen KD, et al. (2009) Gravitational and magnetic separation in self-assembled clay-ferrofluid nanocomposites. Braz. J. Phys. 39: 163-170.

[4] Chantrell RW, Popplewell J, Charles SW, (1978) Measurements of particle size distribution parameters in ferrofluids. IEEE Trans. Magn. 14: 975-977.
